# Solvent- and catalyst-free bio-conversion of waste polyurethane foams into high-performance 3D printing resin

**DOI:** 10.1093/nsr/nwaf501

**Published:** 2025-11-17

**Authors:** Xiaoyu Zhang, Xingqun Pu, Lianlian Xia, Wenjun Peng, Ying Ji, Jieyang Xu, Jingjun Wu, Long Jiang, Qian Zhao, Zizheng Fang, Tao Xie

**Affiliations:** State Key Laboratory of Chemical Engineering and Low-carbon Technology, College of Chemical and Biological Engineering, Zhejiang University, Hangzhou 310058, China; State Key Laboratory of Chemical Engineering and Low-carbon Technology, College of Chemical and Biological Engineering, Zhejiang University, Hangzhou 310058, China; Zhejiang Key Laboratory of Intelligent Manufacturing for Functional Chemicals, ZJU-Hangzhou Global Scientific and Technological Innovation Center, Zhejiang University, Hangzhou 311215, China; Ningbo Innovation Center, Zhejiang University, Ningbo 315807, China; School of Materials Science and Engineering, Zhejiang Sci-Tech University, Hangzhou 310018, China; Key Laboratory of Refrigeration and Cryogenic Technology of Zhejiang Province, Institute of Refrigeration and Cryogenics, Zhejiang University, Hangzhou 310027, China; Key Laboratory of Special Functional and Intelligent Polymer Materials, College of Chemistry and Chemical Engineering, Northwestern Polytechnical University, Xi’an 710129, China; Ningbo Innovation Center, Zhejiang University, Ningbo 315807, China; Key Laboratory of Refrigeration and Cryogenic Technology of Zhejiang Province, Institute of Refrigeration and Cryogenics, Zhejiang University, Hangzhou 310027, China; State Key Laboratory of Chemical Engineering and Low-carbon Technology, College of Chemical and Biological Engineering, Zhejiang University, Hangzhou 310058, China; State Key Laboratory of Chemical Engineering and Low-carbon Technology, College of Chemical and Biological Engineering, Zhejiang University, Hangzhou 310058, China; Zhejiang Key Laboratory of Intelligent Manufacturing for Functional Chemicals, ZJU-Hangzhou Global Scientific and Technological Innovation Center, Zhejiang University, Hangzhou 311215, China; State Key Laboratory of Chemical Engineering and Low-carbon Technology, College of Chemical and Biological Engineering, Zhejiang University, Hangzhou 310058, China

**Keywords:** polyurethane foams, upcycling, dynamic polymer networks, 3D printing, bio-based

## Abstract

The end-of-life treatment of thermoset polyurethane foam (PUF) is an environmental challenge that has remained unresolved for decades. Current chemical recycling strategies are economically prohibitive to implement due to the complex multi-step process and use of catalyst/solvent. Here, we report a one-step upcycling strategy that transforms PUF waste into 3D photo-printing resins without using any catalyst or solvent. Our process employs bio-based itaconic acid as a single reagent that not only degrades PUF into fragments but also generates photocurable vinyl end-groups *in situ*. Incorporation of additional bio-based monomers (tetrahydrofurfuryl methacrylate and l-lysine diisocyanate) allows formulation of a photo-sensitive resin. The corresponding 3D photo-printed polymer, made entirely from PUF waste and bio-monomers (excluding the photo-initiator), exhibits exceptional mechanical performance (tensile strength: 26.3 MPa, toughness: 16.2 MJ m^−3^) relative to other commercial 3D photo-printed polymers. Our simple process that upcycles polymer wastes using solely bio-derived reagents points to a promising direction towards sustainable development of polymer materials.

## Teaser

This paper reports a solvent- and catalyst-free one-step approach that transforms polyurethane foam waste into 3D printing photo-resins using biobased reagents.

## INTRODUCTION

The versatility of urethane chemistry has made polyurethane indispensable for many industrial sectors including automotive, construction and medical devices [1,2]. Among the product variants, thermoset polyurethane foam (PUF) is predominant and accounts for approximately 67% of total production. Due to its low density, the volumetric global annual output of PUF stands at an astonishing 400 million cubic meters. The chemical crosslinked architecture is key to the superior performance of PUF, but it inherently makes recycling challenging. Consequently, most after-service PUF is landfilled or incinerated, causing both environmental pollution and resource waste [3,4]. Physical recycling uses mechanically ground PUF as filler additives; while straightforward, the approach carries low economic returns [5]. For decades, the search for environmentally sustainable and economically feasible recycling strategies has been the focus of extensive industrial and academic research.

Chemical recycling has gained significant attention for its potential to selectively reconfigure the molecular architectures in dealing with the polymer waste streams [6–11]. Conventional approaches for PUF chemical recycling primarily target the cleavage of urethane, urea and biuret linkages through hydrolysis, alcoholysis, glycolysis or acidolysis, aiming to recover the polyol for PUF reconstruction [12–14]. However, the process necessitates high-cost separation/purification steps and achieves only partial monomer recovery, significantly compromising the economic feasibility required for industrial implementation. Recent advancements demonstrate the transformation of PUF into reprocessable dynamic covalent networks by incorporating a catalyst that activates the carbamate exchange reaction, which allows foam-to-foam closed-loop recycling via extrusion of catalyst-loaded PUF granules and blowing agents [15,16]. Foam to foam recycling is also possible by introducing a monomer with more readily dynamic bonds [17] or employing an external reagent that can participate in the network reconfiguration [18]. More recently, upcycling strategies that transform PUF into high-value products have emerged as a promising direction [19,20]. In particular, our group has demonstrated the upcycling of PUF into 3D photo-printable resin [20]. This approach achieves full material recycling without requiring costly purification, and the regenerated 3D-printing resin has significant economic value gain.

Despite progress in the right direction, our previous work employs catalyst and/or solvent [19,20], which present additional challenges. First, these processes often consist of multiple steps that add costs [19,20]. Second, the removal of solvent from the upcycled product(s) is an energy-intensive process [20], and any residual, even if it is minute, would negatively impact the consumer safety of the recycled product(s). Third, the use of a high-loading catalyst [20] is not only cost-prohibitive but also compromises product performance if it is not removed completely. These issues collectively compromise the economic and technological feasibility. To advance PUF recycling technologies for large-scale implementation, an ideal process must simultaneously address environmental sustainability, economic viability and operational simplicity.

Herein, we report a one-step upcycling strategy that meets this ambitious goal. Without using any solvent or catalyst, our process employs bio-based itaconic acid (IA) to not only deconstruct the PUF network into reworkable forms but also functionalize the obtained products in one reaction step. With the addition of bio-based tetrahydrofurfuryl methacrylate (THFMA), l-lysine diisocyanate (LDI), a 3D photo-printable resin, is formulated. Excluding the photo-initiator, the optimal formulation consists entirely of PUF waste (45%) and bio-derived monomers (55%), yet the corresponding 3D photo-printed polymer exhibits exceptional mechanical performance (modulus: 333.3 MPa; tensile strength: 26.3 MPa; toughness: 16.2 MJ m^−3^). In general, the sustainable development of polymer materials is achievable independently via either polymer recycling or making polymers from bio-renewables. Our process uniquely combines both, achieving upcycling of polymer wastes using bio-derived reagents. In addition, accessing high-performance 3D printing resins using polymer waste with only one simple reaction step provides an unanticipated benefit for future flexible manufacturing.

## RESULTS AND DISCUSSION

**Figure 1.**
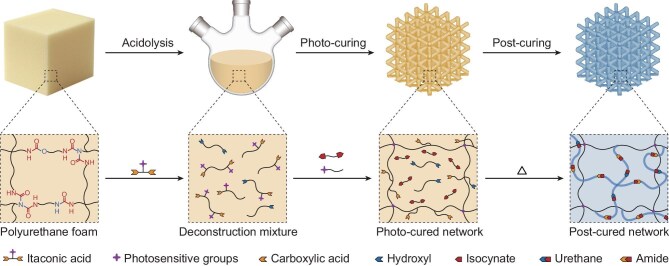
The process and mechanism for upcycling PUFs into a 3D printable network via acidolysis and a dual-curing strategy. Note: urea is also produced in the network due to the reaction between the isocyanate and water in the environment. For simplicity only, it is not shown in the scheme.

Figure 1 illustrates the upcycling strategy for converting commercial thermoset PUF into 3D photo-printable resins. The PUF matrix contains three types of dynamic covalent bonds (urea/urethane/biuret) [18–20], which are targeted for network deconstruction through selective bond cleavages. IA, an α,β-unsaturated dicarboxylic acid, is selected as the acidolysis reagent. This is based on three considerations. First, IA is a low-cost bio-derived chemical. Second, it allows incorporation of photo-active vinyl groups [21–23] into the deconstruction mixture, which are prerequisites for 3D photo-printing. Third, the acidolysis suppresses the formation of amines, which would otherwise consume the vinyl groups via aza-Michael reactions. Following the acidolysis step, two other bio-based monomers, THFMA and LDI, are introduced into the acidolyzed mixture from PUF to form a photo-sensitive resin. Here, THFMA acts as a reactive diluent to reduce the resin viscosity for 3D printing and also participates in the light-triggered radical copolymerization to form a primary photo-printed network. Subsequently, in a post-printing thermal post-curing step, LDI reacts with hydroxyl and carboxylic acid groups. This establishes a secondary interpenetrating network through urethane/amide bond formation [24]. The dual-network architecture, with designable hierarchical hydrogen bonds, can be tailored to access high mechanical performance (Fig. 1). We note that other reactive diluents may also be used. THFMA is selected based on the following considerations. First, THFMA is a bio-based monomer with relatively low viscosity. Second, as a monofunctional monomer, THFMA imparts enhanced mechanical properties to the photocured materials, since the mechanical performance, particularly the breaking strain, is highly sensitive to the content of difunctional or multifunctional monomers in the photocurable system [25].

**Figure 2.**
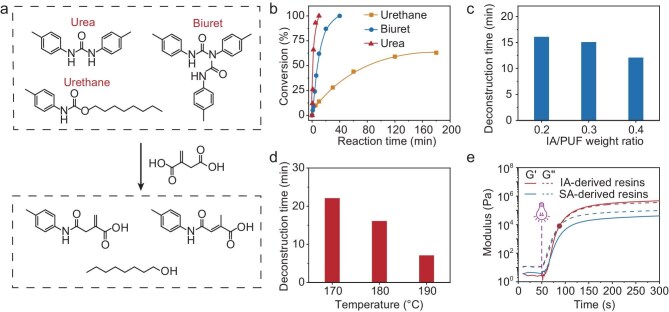
Chemical deconstruction of PUF via acidolysis using IA. (a) Summary of the model compound reactions. (b) Reaction kinetics between IA and biuret/urea/urethane in the model compound experiments. (c) The impact of the IA/PUF weight ratio on the deconstruction time. (d) The influence of the deconstruction temperature on deconstruction time. (e) Photo-rheological curves of IA-derived and SA-derived resins.

To explore the chemical deconstruction mechanism of PUF via acidolysis using IA, model compound experiments are conducted using three molecules containing a urea bond, a biuret bond and a urethane bond, respectively. The synthesis and characterization of these three molecules are provided in our early work [20]. These model molecules are separately reacted with IA at a molar ratio of 1:20 at 180°C. The reactions are monitored by proton nuclear magnetic resonance (^1^H NMR) analyses. The evolution of the ^1^H NMR spectra (Figs S1–S3) suggests that the urea and biuret are converted into carboxylic acids, whereas the urethane is transformed into a carboxylic acid and an alcohol (Fig. 2a). The ^1^H NMR analyses further allow calculation of the reaction conversions as a way to monitor the reaction kinetics. The results as presented in Fig. 2b show that the urea and biuret bonds are notably more reactive than urethane bonds. Under the same reaction conditions, the former two were fully consumed within 40 min, whereas only 30% of the urethane bonds reacted. Prolonging the reaction time allows the urethane to continue reacting, but the conversion is only 63%, even after 180 min (Fig. 2b).

Guided by the above bond cleavage chemistry, we proceed to investigate the chemical deconstruction of PUF. The PUF used here is a commercial soft foam corresponding to a crosslinked network with urea, urethane and biuret bonds at a molar ratio of 3:1:0.2, as determined in our previous work [20]. The PUF is first ground into powders and mixed with IA. Heating induces acidolysis to deconstruct the PUF network. As the reaction proceeds, the PUF powders transform from a solid form to a liquid form at a particular time, which is defined here as the deconstruction time. Two parameters govern the deconstruction process here: the IA/PUF weight ratio and deconstruction temperature. As revealed in Fig. 2c, at a constant deconstruction temperature of 180°C, increasing the IA/PUF weight ratio reduces the deconstruction time. However, such a reduction is relatively small, from 16 min (IA/PUF weight ratio of 0.2) to 12 min (IA/PUF weight ratio of 0.4). Importantly, at an IA/PUF weight ratio of 0.1, the PUF cannot be completely liquified, even after prolonging the reaction time to 3 h (Fig. S4), indicating a threshold weight ratio for effective bond cleavage. During the deconstruction reaction, IA attacks the biuret crosslinks and two other dynamic bonds (urethane and urea) in the network. The reaction kinetics are not only affected by the bond concentrations in the network, but also strongly impacted by the interfacial contact between the liquid melted IA and the solid PUF. The experimentally observed threshold weight ratio reflects the complex interplay between the interfacial contact and the concentrations of cleavable bonds in the PUF network structure. Nevertheless, at a fixed IA/PUF weight ratio of 0.2, the deconstruction time gradually decreases from 22 to 7 min as the temperature increases from 170°C to 190°C (Fig. 2d). The observed lower temperature limit is 165°C, corresponding to the melting point of IA, below which the deconstruction becomes kinetically hindered due to the restricted molecular diffusion. We note that, although 16 min is long enough for PUF deconstruction at 180°C, the viscosity of the obtained mixture is high when it is cooled down to ambient temperature. This makes it practically difficult to ensure a homogeneous mixture with other monomers in formulating the photo-curable resin. Prolonging the deconstruction time reduces the molecular weight of the deconstruction mixture (Table S1). In principle, lower molecular weight favors better printability due to the lower viscosity. In reality, however, the resin printability and the mechanical properties of printed parts are strongly coupled and determined by both the deconstruction mixture and the additional bio-based monomers. Consequently, a standard deconstruction condition (IA/PUF weight ratio of 0.2, 180°C, 1 h) is established here as the deconstruction protocol for further investigation.

According to the reaction pathway (Figs 1 and 2a), the acidolytic deconstruction of the PUF network yields α,β-unsaturated carboxylic acid, which possesses polymerizability with its vinyl moieties. However, the intrinsic reactivities of these α,β-substituted vinyl groups towards photoinduced radical polymerization are too low for 3D photo-printing (Fig. S5). Also, the deconstruction mixture cannot be directly used as photo-polymer resins due to its high viscosity (11 719 Pa·s at ambient temperature at the shear rate of 1 s^−1^; see Fig. S6). In addition, direct use of the deconstructed mixture for 3D printing does not allow tuning of the structure of the polymerized network for potential access to high-performance materials. To address these limitations, a dual-curing photo-resin system is formulated by incorporating additional THFMA and LDI, which are both bio-based. THFMA reduces the viscosity and offers high reactivity towards photo-initiated radical polymerization. Its copolymerization with the deconstructed liquid compensates for the low reactivity of the latter to form a primary photo-cured network. Meanwhile, LDI can react with hydroxyl/carboxyl groups to form a secondary network via post-printing thermal curing (Fig. 1).

Despite their low reactivity, the presence of the α,β-substituted vinyl groups in the deconstruction mixture is crucial for the photo-curing. The ^1^H NMR analysis (Fig. S7) reveals that while elevated deconstruction temperatures (180°C) induced partial vinyl group consumption, the residual equilibrates at a high value of 0.55 mmol/g. We note that IA can undergo isomerization at 180°C to generate citraconic acid and mesaconic acid by conjugated diene formation [26,27] (Fig. S8). However, the photo-curability is maintained as both the isomers retain α,β-unsaturated diacid configurations. To validate the photo-reactivity, a photo-sensitive resin is formulated by mixing the deconstruction mixture (54%), THFMA (30%) and LDI (16%). This formulation is used as a representative unless otherwise noted. Photo-rheologic measurements show that the G′ exceeds G′′ upon photo-illumination at around 37 s, confirming network formation (Fig. 2e). By comparison, a control experiment using succinic acid (SA) as the acidolysis reagent for PUF deconstruction (SA/PUF weight ratio of 0.2, 180°C, 1 h) yields an oligomer without polymerizable moieties. Under an identical formulation protocol, the SA-derived resins exhibit no photo-gelation, as implied by the observation that G′′ is above G′ throughout the experiment (Fig. 2e and Fig. S9). These results verify the essential role of α,β-unsaturated groups in IA, despite their relatively low homo-polymerizability.

**Figure 3.**
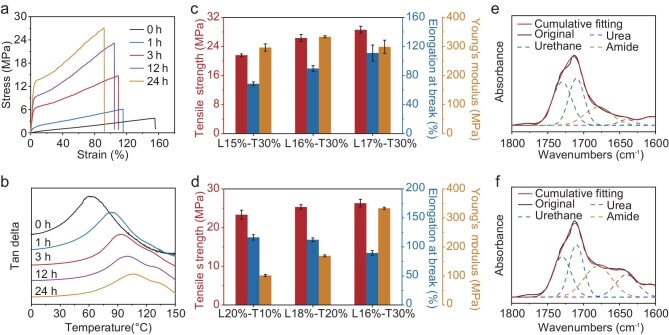
Evolution of thermomechanical and structural characteristics during thermal post-curing. (a) Stress–strain curves with varying post-curing times. (b) DMA curves with varying post-curing times. (c and d) Evolution of tensile strength, elongation at break, and Young’s modulus for samples Lx%-Ty%. (e and f) Peak deconvolution of the C=O band before (0 h) and after (24 h) post-curing.

Having optimized the PUF deconstruction chemistry and formulated the photo-sensitive resin, polymer networks are constructed via a photo-thermal dual-curing strategy. This approach involves sequential photo- and thermo-curing, designed to establish an interpenetrating network architecture to enhance the mechanical properties. Here, a series of samples (see Table S2) are made, denoted as Lx%-Ty%, with x and y corresponding to the weight percentages of LDI and THFMA in the photo-resin, respectively. To evaluate the influence of thermal post-curing on the thermomechanical performance, we focus on a resin formulation of L16%-T30%. The initial photo-cured sample is a soft material with Young’s modulus and tensile strength of 3.5 and 3.5 MPa, respectively (Table S3). Upon thermal post-curing at 90°C, both Young’s modulus and tensile strength are markedly increased (Fig. 3a), reaching maximum values of 333.3 and 26.3 MPa at an optimal post-curing time of 24 h. The corresponding tensile toughness is 16.2 MJ m^−3^. Dynamic mechanical analysis (DMA) curves in Fig. 3b show that the initial state exhibits a glass transition temperature (*T*_g_) of 62°C, which is increased to 106°C after post-curing for 24 h. These changes in macroscopic properties are attributed to the formation of a new network structure during thermal post-curing (Fig. 1). The gel fraction measurement further confirms network formation, increasing from 59.2% after photo-curing to a plateau value of 91.3% upon post-curing for 24 h (Fig. S10). This trend aligns with a concurrent rise in crosslinking density from 103.7 to 742.3 mol m^−3^ (Table S4).

In the deconstruction mixture, the total concentration of reactive hydrogens from the carboxyl and alcohol end-groups is determined as 2.38 mmol/g via isocyanate titration (Fig. S11). This establishes the basis for optimizing the LDI content. While fixing the THFMA content at 30% in the photo-resins, changing the PUF deconstructed mixture and LDI allows tuning of the network to access mechanically distinctive materials. Figure 3c shows that when the isocyanate and reactive hydrogens are maintained at a stoichiometric balance for L15%-T30% the tensile strength and modulus after post-curing are 21.6 and 296.1 MPa, respectively. By comparison, a slight excess of isocyanate in L16%-T30% (molar ratio of 1.1 between isocyanate and reactive hydrogens) yields notably higher tensile strength (26.3 MPa) and modulus (333.3 MPa). This is because a small amount of the isocyanate is inevitably consumed by water to form urea linkages during post-curing [24]. However, an overabundance of isocyanate for L17%-T30% (molar ratio of 1.2 between isocyanate and reactive hydrogens) leads to a comparable tensile strength (28.6 MPa) but lower modulus (298.5 MPa). As for the THFMA (at the fixing molar ratio of 1.1 between isocyanate and reactive hydrogens), Fig. 3d shows that as its content in the photo-resin increases, the modulus after post-curing increases proportionally from 101.6 MPa (L20%-T10%) to 333.3 MPa (L16%-T30%). Its tensile strength follows a similar trend, but within a narrow range between 23.3 and 26.3 MPa. The mechanical properties of the deconstructed PUF mixtures with different deconstruction times are also investigated (Fig. S12). Following photo-curing, both the tensile strength and modulus decrease with prolonged deconstruction times, which is attributed to the increasing content of non-photocurable alcohol. After thermal post-curing, however, longer deconstruction times enhance the mechanical properties, resulting from the significant formation of a secondary polyurethane network via reaction with isocyanate.

The network structural evolution during thermal post-curing, which is key to the mechanical performance of the polymer, is monitored by Fourier transform infrared (FTIR) analyses (Fig. 3e and f). As post-curing proceeds, the characteristic isocyanate peak at 2250 cm^−1^ gets progressively weakened and vanishes after 24 h, indicating its complete consumption (Fig. S13). This observation is consistent with the optimal post-curing times for mechanical enhancement (Fig. 3a). To elucidate the concurrent chemical transformations, the C=O bands in the range of 1800–1600 cm^−1^ are deconvoluted into four subpeaks ascribed to urethane (1710 and 1730 cm^−1^), amide (1680 cm^−1^) and urea (1640 cm^−1^). The result (Fig. 3e) suggests that the as-cured sample contains predominantly amide and urea/urethane bonds. After post-curing, a notable increase in the amide peak intensity is observed (Fig. 3f), verifying the reaction between isocyanate and carboxylic acid. In addition, the concurrent increase in the urea peak intensity indicates the occurrence of reactions between isocyanate and water. Collectively, the urethane, urea and amide bonds establish hierarchical hydrogen bonding interactions in the network, responsible for the observed mechanical strengthening.

**Figure 4.**
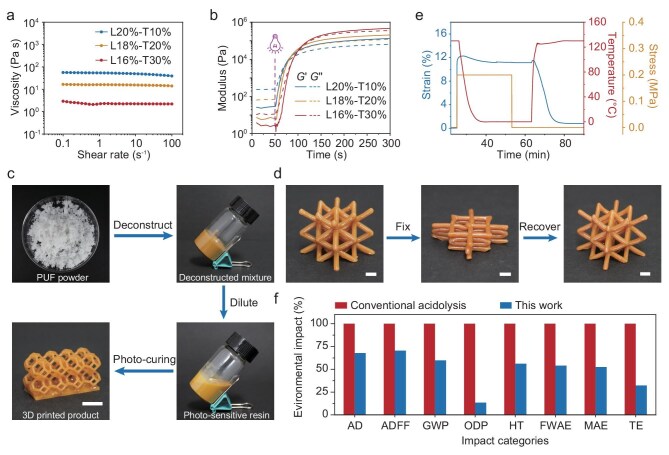
Upcycling PUF waste into DLP photo-resin. (a) The viscosity of the DLP precursor. (b) Photo-rheology measurements of the DLP precursor. (c) Illustration of the PUF upcycling into a DLP precursor and a 3D printed product. (d) Demonstration of the shape-memory process of the 3D printed lattice. (e) Quantitative shape-memory cycle. (f) Normalized comparison of environmental impacts between conventional acidolysis by He *et al.* [13] and this work. AD, abiotic depletion; ADFF, abiotic depletion (fossil fuel); GWP, global warming potential; ODP, ozone layer depletion; HT, human toxicity; FWAE, fresh water aquatic ecotoxicity; MAE, marine aquatic ecotoxicity; TE, terrestrial ecotoxicity. Scale bar: 0.5 mm.

The resin formulation has a strong impact on 3D printing, particularly the viscosity and the photo-polymerization kinetics. Increasing the THFMA content reduces the viscosity (Fig. 4a), from 54.13 Pa·s for L20%-T10% to 2.27 Pa·s for L16%-T30%. The latter is well suited for digital light processing (DLP) printing (<5 Pa·s) [28,29]. The photopolymerization kinetics of different formulations are examined by photo-rheological analysis, which monitors the change in storage moduli (G′) and loss moduli (G′′). Upon UV exposure, both moduli exhibit rapid increases for all the formulations, with the gelation point (determined as the intercept points between G′ and G′′) occurring upon photo-illumination at 37 s for L16%-T30%. The longer gelation time corresponding to a higher THFMA content is attributed to the reduced concentration of the crosslinking agent derived from the deconstruction mixture. The L16%-T30% formulation displays the most drastic G′ increase and the highest plateau modulus, suggesting the effective integration of THFMA into the photo-cured network. In addition, FTIR analysis shows a double bond conversion of approximately 80.2%, calculated from the reduction of the characteristic peak at 815 cm^−1^ before and after photo-curing (Fig. S14). With the above results, L16%-T30% is selected for 3D printing due to its low viscosity, optimal gelation kinetics and enhanced mechanical performance. Moreover, the resin exhibits high storage stability, as evidenced by the well-preserved isocyanate functionality (Fig. S15) even after storage for 30 h (ambient temperature under dark conditions). Figure 4c demonstrates visually the transformation of waste PUF into a photo-resin that is subsequently printed into a geometrically complex 3D object. The mechanical performance (tensile strength: 26.3 MPa, toughness: 16.2 MJ m^−3^) of our 3D-printed products surpasses most commercial 3D photo-printed products (Fig. S16, references provided in the Supplementary data), making it practically suitable for mechanically demanding applications.

The post-cured 3D printed polymer, with a *T*_g_ of 106°C, shows shape-memory characteristics. The 3D-printed lattice (Fig. 4d) can be fixed into a temporary shape (0°C) and recovered to the original one by heating (130°C). The corresponding shape fixing and recovery rates calculated from the quantitative shape-memory cycle (Fig. 4e) are 90.0% and 92.6%, respectively. This highlights the possibility of turning the PUF waste into value-added functional devices.

The impact of our strategy for PUF recycling on sustainability is evaluated by conducting life cycle assessment (LCA), using conventional acidolysis recycling [13] as the baseline comparison. The LCA results (details presented in Table S5) reveal that our process exhibits a better overall environmental impact, with notably lower normalized results in abiotic depletion, abiotic depletion (fossil fuel), global warming potential, ozone layer depletion, human toxicity, fresh water aquatic ecotoxicity, marine aquatic ecotoxicity and terrestrial ecotoxicity (Fig. 4f).

## CONCLUSION

In summary, we have reported a solvent-free, catalyst-free, one-step strategy for simultaneous deconstruction and upcycling of PUF into high-performance, bio-based photo-curable resins suitable for digital light 3D printing. This approach facilitates the creation of an interpenetrating network architecture via sequential photo-thermal dual curing. The optimized resin consists entirely of PUF waste (45%) and bio-derived monomers (55%), yet the printed material shows exceptional mechanical performance with a tensile strength of 26.3 MPa and toughness of 16.2 MJ m^−3^. In addition, the upcycled 3D-printed product exhibits shape-memory characteristics for smart material development, highlighting the potential of sustainable 3D printing technologies for next-generation manufacturing.

## Supplementary Material

nwaf501_Supplemental_File
