# Supplementary material for: Solvent- and catalyst-free bio-conversion of waste polyurethane foams into high-performance 3D printing resin
Source: Natl Sci Rev. 2025 Nov 17;12(12):nwaf501. doi: 10.1093/nsr/nwaf501 (PMC12715862; doi:10.1093/nsr/nwaf501)
Supplement: nwaf501_Supplemental_File [file nwaf501_supplemental_file.pdf]

Solvent- and catalyst-free bio-conversion of waste polyurethane foams into high performance 3D printing resin

Xiaoyu Zhang[1#], Xingqun Pu[1,2#], Lianlian Xia[3], Wenjun Peng[4], Ying Ji[5], Jieyang Xu[6], Jingjun Wu[3], Long Jiang[5], Qian Zhao[1], Zizheng Fang[1,2]*, Tao Xie[1]*

[1]State Key Laboratory of Chemical Engineering and Low-carbon Technology, College of Chemical and Biological Engineering, Zhejiang University, Hangzhou, 310058, P. R. China
[2]Zhejiang Key Laboratory of Intelligent Manufacturing for Functional Chemicals, ZJU-Hangzhou Global Scientific and Technological Innovation Center, Zhejiang University, Hangzhou, 311215, P. R. China
[3]Ningbo Innovation Center, Zhejiang University, Ningbo, 315807, China
[4]School of Materials Science and Engineering, Zhejiang Sci-Tech University, Hangzhou, 310018
[5]Key Laboratory of Refrigeration and Cryogenic Technology of Zhejiang Province, Institute of Refrigeration and Cryogenics, Zhejiang University, Hangzhou. 310027, China
[6]Key Laboratory of Special Functional and Intelligent Polymer Materials, College of Chemistry and Chemical Engineering, Northwestern Polytechnical University, Xi'an, 710129

[#]These authors contributed equally to this work
*Correspondence: fangzizheng@zju.edu.cn; taoxie@zju.edu.cn

Materials and methods

Materials:

L-lysine diisocyanate (LDI), succinic acid (SA), tetrahydrofurfuryl methacrylate (THFMA), and phenyl bis(2,4,6-trimethylbenzoyl) phosphine oxide (Irgacure 819) were obtained from Macklin. Itaconic acid (IA), *p*-toluidine, *p*-tolyl isocyanate, and 1-octanol were purchased from Aladdin. Commercial polyurethane foams were kindly supplied by UE Furniture. All chemicals were used as received without further purification.

General characterization:

^{1}H NMR analysis was conducted using the 500 MHz Bruker instrument. FTIR spectra were recorded using a Nicolet iS50 instrument (Thermo Fisher) equipped with a diamond-attenuated total reflectance (ATR) attachment. The stress-strain curves were collected using a universal material testing machine (SUNS, 100 N load cell) at a constant stretching speed of 100 mm min^{-1}. The samples were cut into dumbbell shapes (20 × 2 × 0.3 mm^3) before use, and at least three specimens were tested for each sample. The viscosity of the photocurable resin was measured using a rheometer (MARS 60) at a shear rate ranging from 0.1 to 100 s^{-1} at 25 ℃. Photorheology experiments were performed using an Anton Paar MCR 702e rheometer fixed with a detachable photo-illumination system (OmniCure S1500). The storage (G′) and loss (G″) moduli were recorded at a constant frequency of 1 Hz during UV exposure (10 mW cm^{-2}, 300 s). The molecular weight was determined by Gel permeation chromatography (model Waters 1525/2707) using DMF as the eluent (flow rate: 1 mL min^{-1}, 45 °C), with polystyrene as the calibration standard.

Chemical deconstruction of PUF:

In a three-necked flask (500 mL), PUF (30 g) and IA were mixed with mechanical stirring for a preset time and temperature.

Model compound experiments:

Three small-molecule compounds containing urea, urethane, and biuret bonds were synthesized according to previous work [1], and their deconstruction behaviors were

investigated as follows. Excessive IA (20 times the molar mass of the small-molecule compound) was heated at 180 ℃ for 30 minutes until it was totally melted, followed by adding the small-molecule compound for the reaction.

Determination of reactive hydrogen content:

The reactive hydrogen (including carboxyl and alcohol groups) was determined by the consumed isocyanate using FTIR analysis. Specifically, the deconstruction mixture (0.2 g) dissolved in DMF (0.85 mL) was reacted with different amounts of isophorone diisocyanate at 90 ℃ for 24 h. Afterward, the intensity of the isocyanate peak at 2260 cm^{-1} was monitored, and the concentration of reactive hydrogen was calculated by fitting the feeding and remaining isocyanate concentration.

Gel fraction test and the calculation of crosslinking density:

Pre-weighed samples (m_0) were immersed in DMF at room temperature overnight to reach a swollen state (m_s). The swollen samples were then vacuum-dried at 80 ℃ overnight to get the final dried mass (m_d).

The gel fraction (G) was calculated as follows:

$$G=m_d/m_0$$

The swelling ratio (S) was calculated as follows:

$$S=m_s/m_d$$

The crosslinking density (V_e) was calculated using the Flory-Rehner equations [2]:

$$V_e = \frac{-[\ln(1-\mathrm{V}_r) + \mathrm{V}_r + \chi V_r^2]}{V_0\left(\mathrm{V}_r^{\frac{1}{3}}\right)}$$

In this equation, V_r is the polymer volume fraction, calculated by:

$$V_r = \frac{\frac{m_d}{\rho_2}}{\frac{m_d}{\rho_2} + (m_s - m_d)/\rho_1}$$

Where V_0 and χ represent the molar volume of DMF (7.74 × 10^{-5} m^3/mol) and the Flory interaction parameter (0.2) [3], while ρ_1 and ρ_2 are the density of DMF (0.944 × 10^6 g/cm^3) and polymer (1.000 × 10^6 g/cm^3).

Preparation of photocurable resins for 3D printing:

The deconstruction mixture was mixed with THFMA and ultrasonicated at room temperature for 60 min, using an ultrasonic cleaner (Model SN-QX-32D) operating at 40 kHz. Subsequently, LDI and photoinitiator were added under continuous mechanical stirring at room temperature to ensure uniform dispersion. Printing was conducted using a top-down DLP 3D printer (DLP DM200, 14 mW cm^{-2}), with an exposure time of 30 s for each layer. The printed objects were post-cured in a UV chamber for 120 s (IntelliRay 600 Flood UV, Uvitron International, 955 mW cm^{-2}, 265~700 nm), followed by thermally post-curing at 90 ℃.

LCA characterization:

To evaluate the potential value of our chemical upcycling strategy towards 3D photo-printing resins, the LCA was adopted here to compare our process with the conventional acidolysis that is widely used for the chemical recycling of commercial PUF (reference process). With different target productions, the system boundary was determined from the waste commercial PUF to resins (our work) and foam (conventional strategy), respectively. The target productions were set with a consistent functional unit of 1 kg. The data relating to the PUF end-of-life processes were derived from the Ecoinvent database (version 3.5) with the CML-IA method in Simapro software, which employed the allocation at point of substitution, cut-off by classification, and consequential data. Besides, life cycle impact assessment converted the process data to environmental information. Herein, the impact categories based on Eco-indicator 99 were chosen, including abiotic depletion, abiotic depletion (fossil fuel), global warming potential, ozone layer depletion, human toxicity, fresh water aquatic ecotoxicity, marine aquatic ecotoxicity, and terrestrial ecotoxicity.

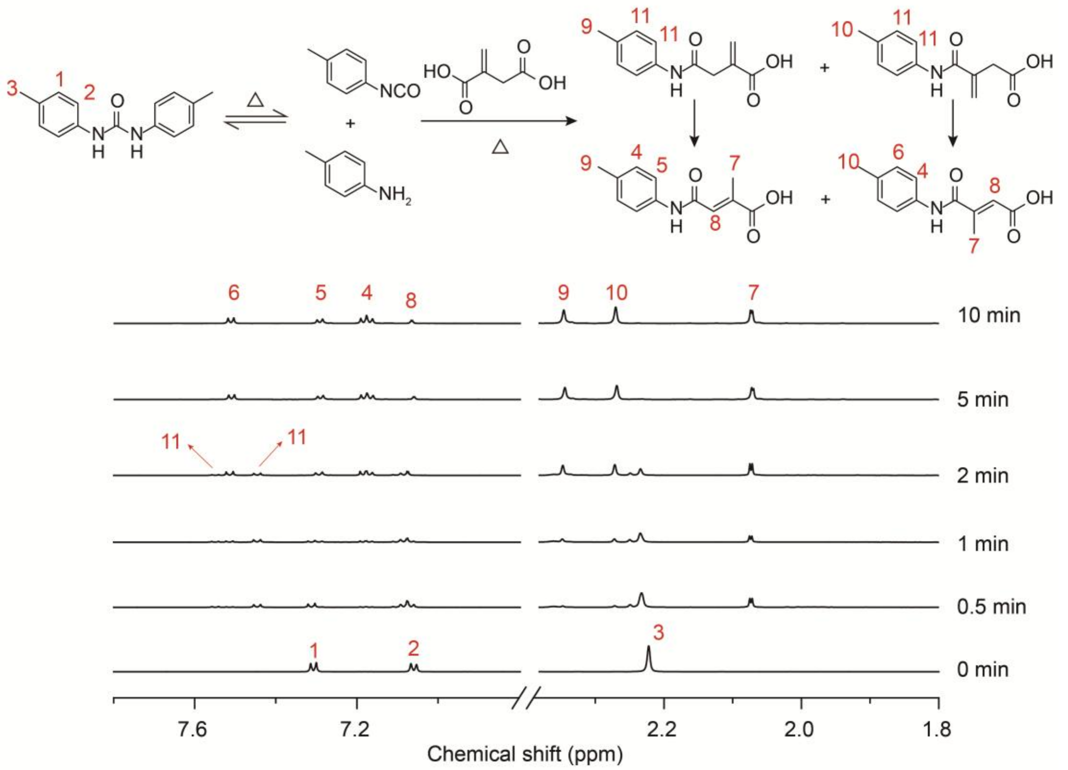

Figure S1. The degradation reaction pathway of IA to 1,3-di-*p*-tolylurea and the ^{1}H NMR spectra at different reaction times.

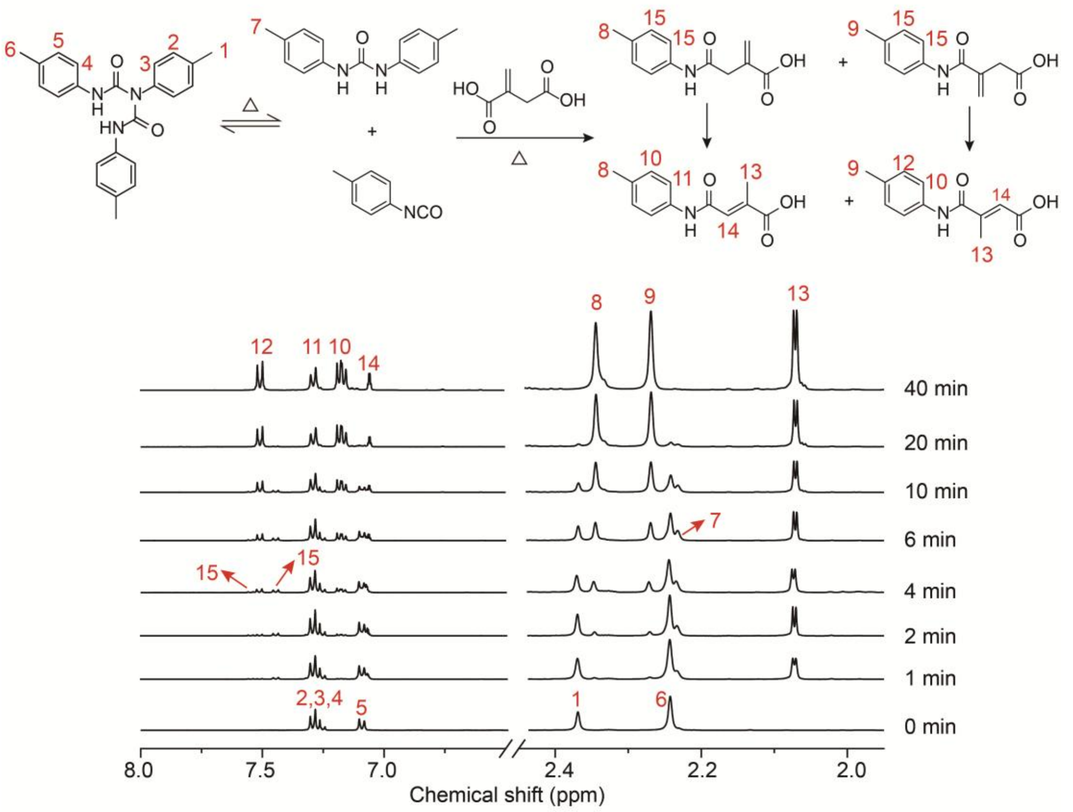

Figure S2. The degradation reaction pathway of IA to 1,3,5-tri-*p*-tolylbiuret and the ^{1}H NMR spectra at different reaction times.

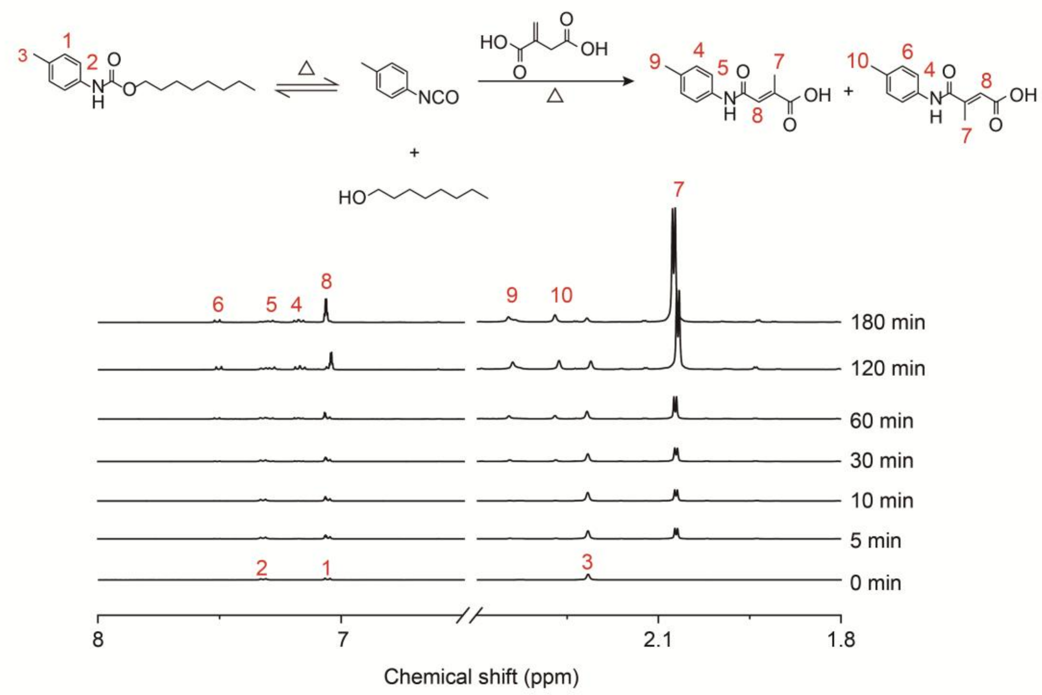

Figure S3. The degradation reaction pathway of IA to octyl-*p*-tolylcarbamate and the ^{1}H NMR spectra at different reaction times.

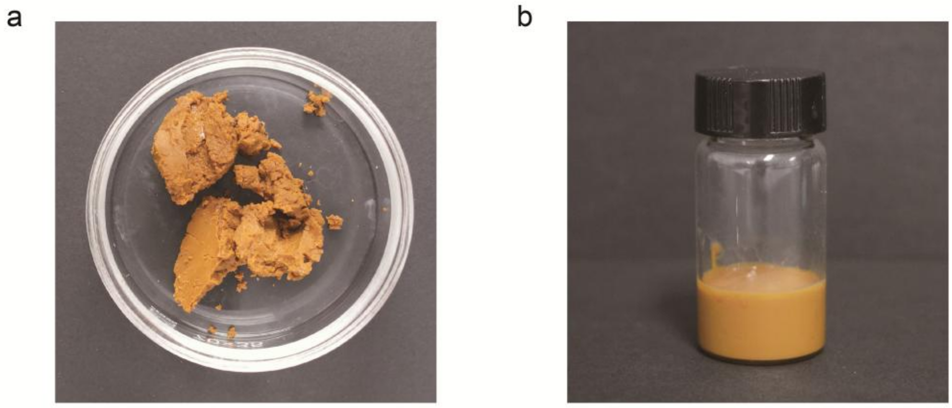

Figure S4. The appearance of the PUF deconstruction mixture after cooling at 25 ℃ with different deconstruction conditions. (a) At the IA/PUF weight ratio of 0.1 after reacting for 3 h. (b) At the IA/PUF weight ratio of 0.2 after reacting for 1 h.

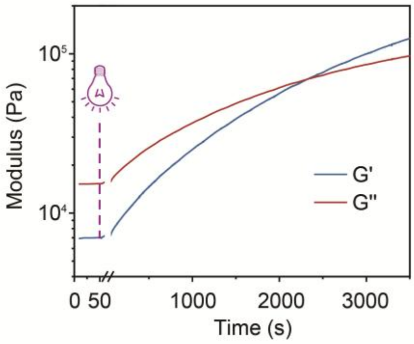

Figure S5. Photo-rheological curve of the PUF deconstruction mixture. The result indicates that the deconstruction mixture is capable of self-polymerizing into a network, albeit requiring a longer exposure time (2272 s).

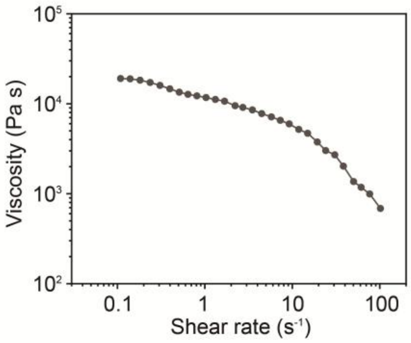

Figure S6. The viscosity of the PUF deconstruction mixture.

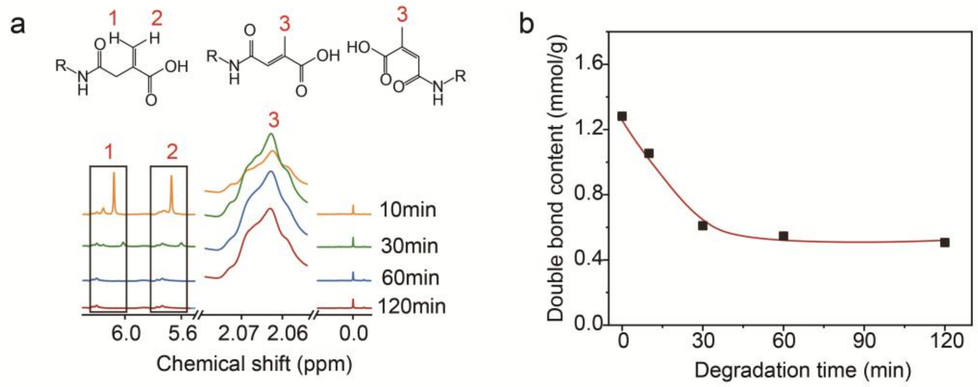

Figure S7. The characterization of vinyl group change during PUF deconstruction. (a) The ^{1}H NMR spectra (DMSO-d6) of deconstruction mixtures for different deconstruction times. (b) The calculated double bond content in deconstruction mixtures varied with deconstruction times.

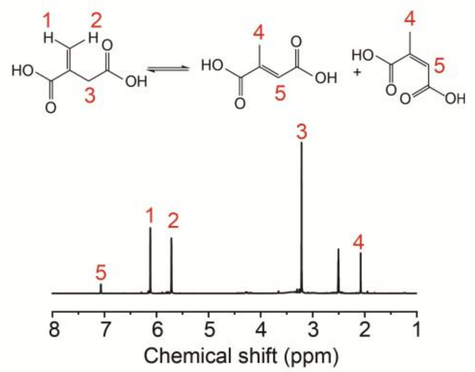

Figure S8. The ^{1}H NMR spectra (DMSO-d6) of IA after thermal annealing at 180 ℃ for 1 h.

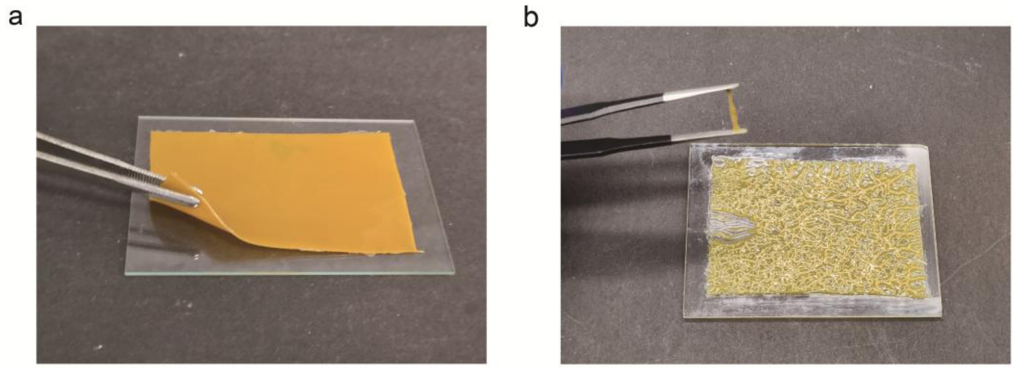

Figure S9. The appearance of the resin after photocuring. (a) IA-derived resin after photo-curing can be peeled as a film with integrity. (b) SA-derived resin after photo-curing is still a viscous liquid.

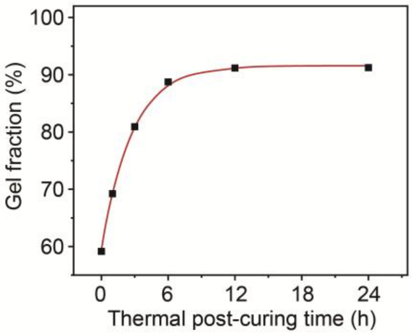

Figure S10. Evolution of the gel fraction with thermal post-curing times.

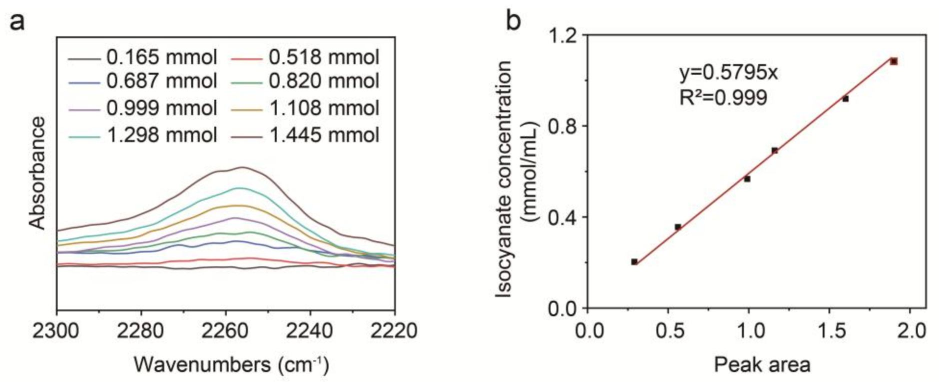

Figure S11. Determination of the reactive hydrogen (carboxyl and alcohol group) in the deconstruction mixture. (a) FTIR spectra of the deconstruction mixture reacting with different amounts of IPDI at 90 ℃ for 24 h. (b) Fitting curve between isocyanate concentration and peak area.

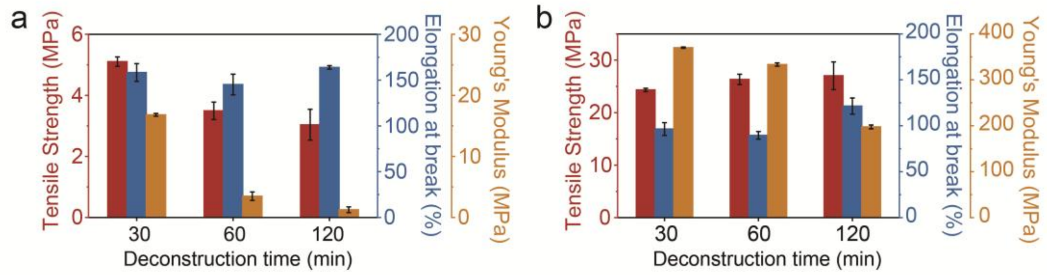

Figure S12. The tensile strength, elongation at break, and Young's modulus of the samples prepared from the resin L16%-T30% with different PUF deconstruction times. (a) Sample before thermal post-curing (0 h). (b) Samples after thermal post-curing (24 h).

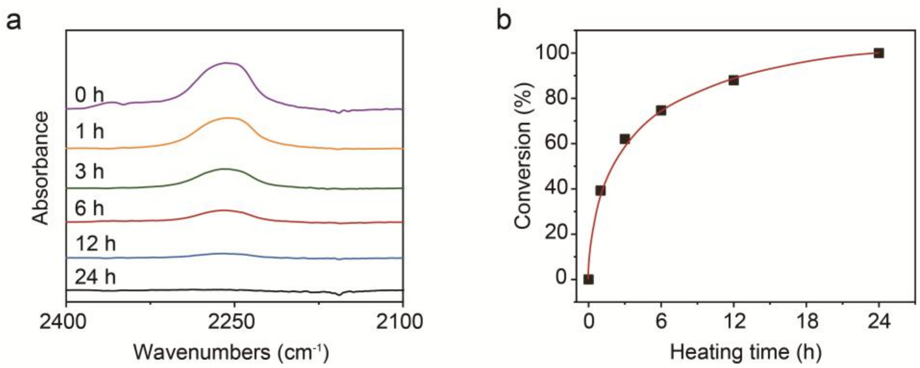

Figure S13. Monitoring the network structural evolution during thermal post-curing. (a) FTIR spectra changed with varying post-curing times in the range of 2400 cm^{-1} to 2100 cm^{-1}. (b) The conversion of the isocyanate calculated from the FTIR result.

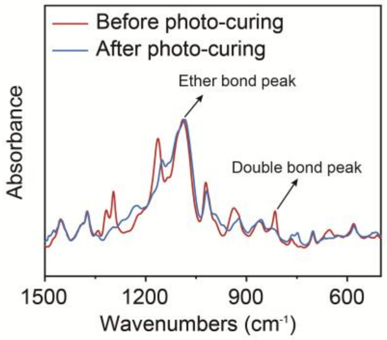

Figure S14. FTIR spectra of the resin L16%-T30% before and after photo-curing. The double bond (815 cm^{-1}) conversion was determined by FTIR spectroscopy using the normalized integrated area of the ether bond (1090 cm^{-1}).

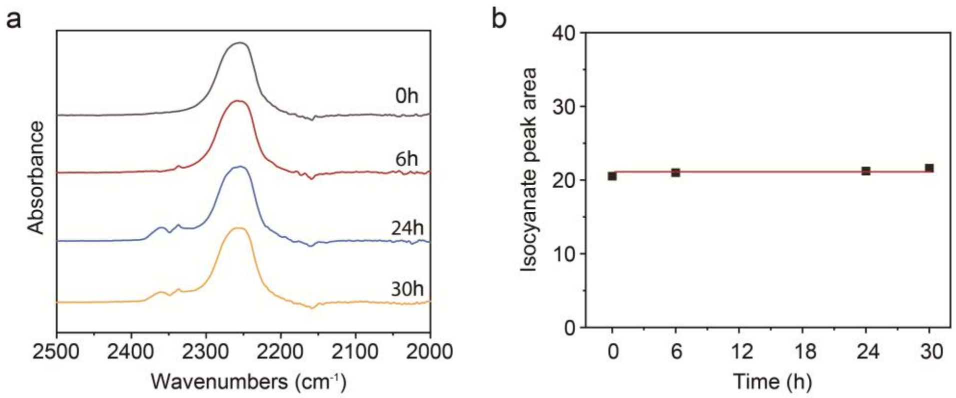

Figure S15. Characterization of the storage stability of the resin by monitoring the isocyanate group change. (a) FTIR spectra of the resin after being stored for different times. (b) The calculated peak area of isocyanate.

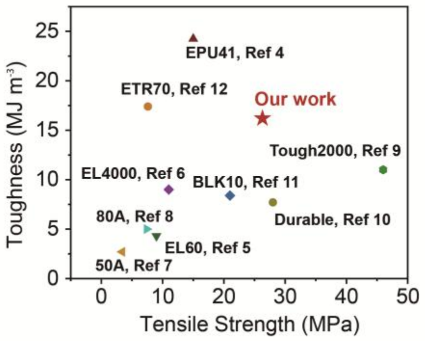

Figure S16. Comparison of the toughness and tensile strength between our material and commercial 3D-printing materials.

Table S1. The molecular weight of the PUF deconstruction mixture with different deconstruction times.

Deconstruction time (min)	M_n	Polydispersity
30	34,293	2.57
60	29,397	2.27
120	26,503	1.61

Table S2. Photo-resin formulations.

Sample	PUF (wt%)	IA (wt%)	THFMA (wt%)	LDI (wt%)
L15%-T30%	46	9	30	15
L16%-T30%	45	9	30	16
L17%-T30%	44	9	30	17
L18%-T20%	52	10	20	18
L20%-T10%	58	12	10	20

Table S3. Mechanical properties of different resin formulations.

Sample	Tensile strength (MPa)		Elongation at break (%)		Young's modulus (MPa)	
	**0 h**	**24 h**	**0 h**	**24 h**	**0 h**	**24 h**
L15%-T30%	3.0±0.2	21.6±0.4	171±4	69±3	1.2±0.2	296.1±12.7
L16%-T30%	3.5±0.3	26.3±1.0	145±11	90±4	3.5±0.7	333.3±3.7
L17%-T30%	2.9±0.2	28.6±1.0	203±7	111±11	0.8±0.1	298.5±23.1
L18%-T20%	2.3±0.3	25.3±0.6	159±5	112±3	0.7±0.1	169.2±3.4
L20%-T10%	1.3±0.1	23.3±1.2	154±4	117±5	0.4±0.1	101.6±3.3

Table S4. Swelling ratio and crosslinking density of samples before (0 h) and after (24 h) thermal post-curing.

Samples	Swelling ratio	Crosslinking density (mol m^{-3})
Before thermal post-curing	9.0 ±0.1	103.7 ±2.1
After thermal post-curing	3.2 ±0.1	742.3 ±10.2

Table S5. LCA results.

Impact categories	Unit	Normalized results in total	
		Our process	Reference process
Abiotic depletion	kg Sb eq	5.57E-5	8.21E-5
Abiotic depletion (fossil fuel)	MJ	105	149
Global warming potential	kg CO_2 eq	7.06	11.8
Ozone layer depletion	kg CFC-11 eq	1.34E-7	9.99E-7
Human toxicity	kg 1,4-DB eq	3.44	6.13
Fresh water aquatic ecotox	kg 1,4-DB eq	2.6	4.81
Marine aquatic ecotoxicity	kg 1,4-DB eq	7820	14900
Terrestrial ecotoxicity	kg 1,4-DB eq	0.0126	0.039

References

1. Liu Z, Fang Z, Zheng N *et al.* Chemical upcycling of commodity thermoset polyurethane foams towards high-performance 3D photo-printing resins. *Nat Chem* 2023; **15**: 1773-1779.
2. Mu H, Sun Z, Chen J *et al.* 3D-printing of ultratough and healable elastomers. *Adv Mater* 2025; **37**: 2507908.
3. Orwoll RA, Arnold PA, Polymer-solvent interaction parameter χ, in: J. Mark (Ed.), *Physical Properties of Polymers Handbook*, Springer, New York, 2007; 233-257.
4. https://learn.carbon3d.com/materials/epu-41
5. https://forward-am.com/material-portfolio/ultracur3d-photopolymers/flexible-elastomeric-line/ultracur3d-el-60/
6. https://forward-am.com/material-portfolio/ultracur3d-photopolymers/flexible-elastomeric-line/ultracur3d-el-4000/
7. https://formlabs.com/store/materials/elastic-50a-resin-v2/
8. https://formlabs.com/store/materials/biomed-flex-80a-resin/
9. https://formlabs.com/store/materials/tough-2000-resin/
10. https://formlabs.com/store/materials/durable-resin/
11. https://www.3dsystems.com/materials/figure-4-rubber-blk-10
12. https://proto3000.com/materials/elastic-toughrubber70/
